# Two delayed-diagnosis case reports of long-lasting thrombocytopenia with splenomegaly

**DOI:** 10.1097/MD.0000000000039354

**Published:** 2024-08-23

**Authors:** Bing Chen, Wenchu Dai, Yuni Xu, Feng Wang, Jinlin Liu

**Affiliations:** a Department of Clinical Laboratory, Wenchang People’s Hospital, Wenchang, Hainan, China; b Department of Clinical Laboratory, Xian No. 1 Hospital, Xian, Shanxi, China; c Department of Laboratory Medicine, The Second Affiliated Hospital of Hainan Medical University, Haikou, China; d Department of Laboratory Medicine, The Affiliated Hospital of Medical School, Ningbo University, Ningbo, China; e Department of Clinical Laboratory, South China Hospital, Medical School, Shenzhen University, Shenzhen, China.

**Keywords:** Gaucher disease, splenomegaly, thrombocytopenia, delayed diagnosis

## Abstract

**Rationale:**

Gaucher disease (GD) is a rare hereditary lysosomal storage disorder disease progression and inappropriate treatment. However, not all patients with GD receive timely diagnosis and treatment.

**Patient concerns:**

Early diagnosis is important for initiating proper treatment and preventing complications.

**Diagnoses:**

Two patients were diagnosed as GD in this study.

**Interventions and outcomes:**

These 2 patients received the imiglucerase enzyme replacement and symptoms significantly improved by the follow-up.

**Lessons:**

Herein, we report 2 patients with a delayed diagnosis of GD to increase awareness and improve education regarding rare diseases. However, noninvasive β-glucocerebrosidase activity or *GBA* gene testing had not been done before bone marrow aspiration, which are the noninvasive and reliable tests that indicate the diagnosis of GD.

## 1. Introduction

Gaucher disease (GD) is a rare autosomal recessive lysosomal storage disease commonly presenting in pediatric patients with massive splenomegaly. It is caused by mutations in the glucocerebrosidase (GBA) gene that eliminate or significantly reduce the activity of β-glucocerebrosidase (GCase), leading to the accumulation of glucocerebroside in macrophages of several organs, mainly the spleen, liver, and bone marrow.^[1]^ The global prevalence of GD ranges from 0.39 to 5.80 per 100,000 persons. However, in China, GD is extremely uncommon with a lower incidence rate than the global average.^[2,3]^GD is classified into 3 types based on clinical manifestations: non-neuronopathic (GD1), acute neuronopathic (GD2), and chronic neuronopathic (GD3). Nearly 95% of GD cases are GD1.^[4]^ Clinically, patients with GD are often misdiagnosed and may experience significantly delayed diagnosis owing to clinical heterogeneity, including hepatosplenomegaly, cytopenia, delayed growth, abdominal pain, chronic fatigue, and neurological impairments such as cognitive decline. Once GD is suspected, it is confirmed by establishing deficient GCase activity and/or *GBA* pathogenic genetic testing.^[5]^ Timely treatment can prevent irreversible damage and improve the quality of life. However, misdiagnosis can lead to disease complications, persistence of untreated symptoms, and depression without an explanation for symptoms.^[2,5,6]^ Moreover, the diagnosis and management of patients with chronic multisystem disorders has evolved substantially in recent years. In this report, we present 2 Chinese female patients who presented with long-lasting splenomegaly and thrombocytopenia but who initially refused to undergo further an invasive bone marrow biopsy at a local hospital. However, after admission to our hospital, invasive bone marrow biopsy clearly showed Gaucher-like cells in these 2 patients, leading to a definitive diagnosis of GD. Therefore, we highlight that diagnostic delays in GD cases can occur owing to the refusal of invasive bone marrow biopsies. Although rare, a diagnosis of GD should be considered in every patient with hepatosplenomegaly and thrombocytopenia. In addition, noninvasive GCase activity and *GBA* gene testing can confirm this rare disease if a patient refuses a bone marrow biopsy. Prompt diagnosis is becoming increasingly important as effective therapies are developed for patients with GD.

## 2. Case presentation

### Case 1:

**Figure 1.**
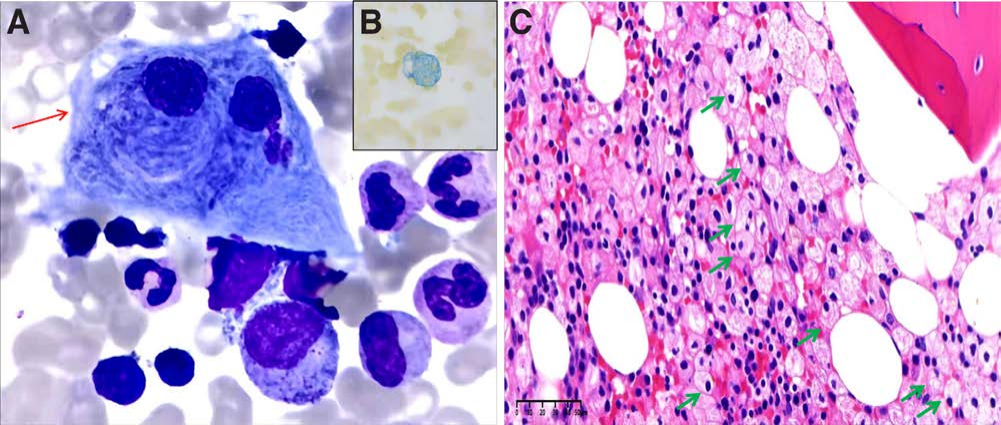
Bone marrow examination in Case 1 of long-lasting splenomegaly and thrombocytopenia. (A) Gaucher-like cells are found on the bone marrow smear (Wright–Giemsa staining, 1000×, red arrow). (B) These cells present green color after ferric staining (1000×), and (C) hematoxylin and eosin (H&E) staining reveals “onion bulb-like” cells (400×, green arrow).

A 30-year-old Chinese female was admitted to our hospital with persistent abdominal distension after eating, which had been ongoing for over a year. Her medical history included thrombocytopenia and mild splenomegaly for nearly 10 years, but she had previously refused an invasive bone marrow biopsy at a local hospital. Laboratory data revealed a total leukocyte count of 3.5 × 10^9^/L, including 68% neutrophils and 24% lymphocytes; hemoglobin, 126 g/L; and platelet count, 81 × 10^9^/L. Ultrasonography confirmed splenomegaly and the patient underwent bone marrow aspiration. The bone marrow smears revealed Gaucher-like cells, comprising 4% of nucleated cells, with a “crumpled tissue paper” appearance in the cytoplasm and 2 eccentric nuclei (Fig. 1A). These cells presented green color after ferric staining (Fig. 1B), and hematoxylin and eosin (H&E) staining revealed onion bulb-like cells (Fig. 1C). LC/MS/MS GCase results indicated a low GCase value of 0.87 μmol/L.h (normal range 1.26–22.23 μmol/L.h) and genetic analysis revealed *GBA* c.454 + 1G > C and c.232C > G (p.Arg 78 Gly) mutations. Hence, a diagnosis of GD was made.

### Case 2:

**Figure 2.**
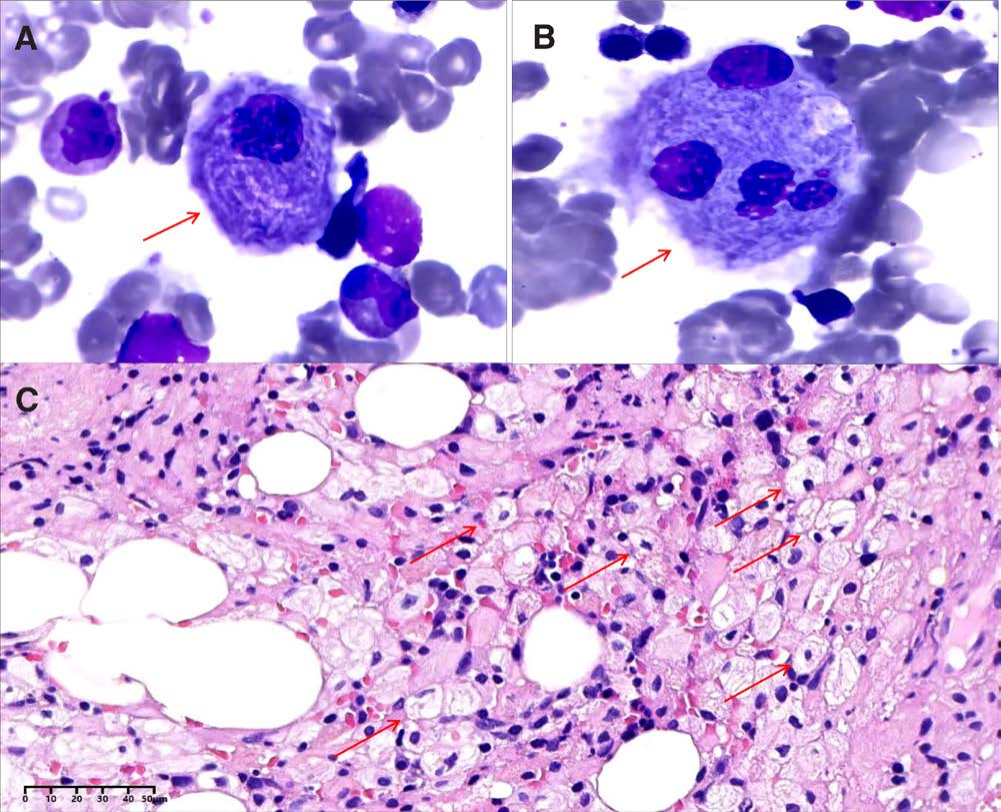
Bone marrow examination in Case 2 of long-lasting splenomegaly and thrombocytopenia. (A and B) The bone marrow aspirate displays cytoplasmic “crumpled tissue paper” morphology with multiple eccentric nuclei (red arrow, 1000×, Wright–Giemsa staining). (C) After H&E staining, these cells display foamy cytoplasm (red arrow, 400×).

A 59-year-old Chinese female admitted to our hospital presented with a history of splenomegaly and thrombocytopenia for 20 years. Living in a rural area, she rarely visited a hospital, and her medical history was insignificant. She treated her thrombocytopenia and easy bruising irregularly with Chinese herbal medicine. Laboratory data revealed a leukocyte count of 2.4 × 10^9^/L, including 73% neutrophils and 22% lymphocytes; hemoglobin, 96 g/L; and a platelet count of 45 × 10^9^/L. Liver and kidney function indices, including alanine transaminase, aspartate aminotransferase, uric acid, and creatinine levels, were within normal reference values. Physical examination and ultrasonography confirmed the presence of splenomegaly. To determine the cause of splenomegaly and thrombocytopenia, a bone marrow aspirate was performed, which revealed Gaucher cells; 5% of the nucleated cells displayed cytoplasmic “crumpled tissue paper” morphology with multiple eccentric nuclei (Fig. 2A and B). After H&E staining, these cells displayed a foamy cytoplasm (Fig. 2C). These Gaucher-like or pseudo-Gaucher cells may also be observed in bone marrow aspirates involving different hematologic disorders; hence, the lysosomal GCase activity and the gene involved in GD were tested at a reference laboratory. The results revealed a low GCase value of 2 nmol/mg.h (normal range 10–25 nmol/mg.h) and 2 pathogenetic *GBA* mutations: c.1240G > C (p.Val 414 Leu) and c.1193G > C (p.Arg 398 Pro). Consequently, a diagnosis of GD was made. Additionally, these 2 patients received the imiglucerase enzyme replacement every 2 weeks after the diagnosis and symptoms significantly improved by the follow up.

## 3. Discussion

Gaucher disease is a lysosomal storage disorder caused by *GBA* gene mutations. Reduced or absent GCase activity results in the accumulation of glucosylceramide and glucosylsphingosine in phagocytes.^[7]^ Typical Gaucher cells presenting with “crumpled tissue paper” or ‘onion bulb’ morphology, can be easily discerned on bone marrow smears to confirm this rare disease. GCase activity and *GBA* gene sequencing can confirm this rare disease, enabling timely diagnosis and treatment. In these 2 cases, we highlight the importance of timely invasive bone marrow biopsy for patients with splenomegaly and thrombocytopenia. Gaucher disease is frequently underdiagnosed but can identified on bone marrow smears. Additionally, GD should be considered in all patients with hepatosplenomegaly and thrombocytopenia. Moreover, both patients described here lived in rural areas, rarely visited hospitals, and their clinical histories were insignificant. One patient had persistent abdominal distension after eating, and the other experienced easy bruising. Notably, both patients were apprehensive about invasive bone marrow aspiration and relied on traditional Chinese medicine or other symptom-relieving drugs instead of opting for bone marrow aspiration to determine the real cause of their symptoms. Their cognitive biases against invasive bone marrow aspiration resulted in a long-standing delayed diagnosis of GD, as well as thrombocytopenia, splenomegaly, abdominal distension, and easy bruising. However, we should bear in mind that noninvasive GCase activity and *GBA* gene testing can confirm this rare disease if the patient refuses an invasive bone marrow biopsy. Our case studies had certain limitations, such as the lack of prior consideration for noninvasive GCase activity or *GBA* gene testing before bone marrow aspiration. This may be due to the disease rarity or insufficient awareness regarding this disorder. In conclusion, noninvasive GCase activity measurement could identify the cause of long-standing splenomegaly and thrombocytopenia. This study highlights the causes of delayed diagnosis of GD in these 2 patients and aims to increase awareness and education concerning rare diseases.

## Author contributions

**Data curation:** Yuni Xu, Feng Wang.

**Formal analysis:** Bing Chen, Wenchu Dai, Yuni Xu, Feng Wang.

**Investigation:** Bing Chen.

**Writing – original draft:** Bing Chen.

**Writing – review & editing:** Jinlin Liu.
